# Influence of zoledronic acid on disseminated tumor cells in bone marrow and survival: results of a prospective clinical trial

**DOI:** 10.1186/1471-2407-13-480

**Published:** 2013-10-15

**Authors:** Malgorzata Banys, Erich-Franz Solomayer, Gerhard Gebauer, Wolfgang Janni, Natalia Krawczyk, Hans-Joachim Lueck, Sven Becker, Jens Huober, Bernhard Kraemer, Birgit Wackwitz, Peter Hirnle, Diethelm Wallwiener, Tanja Fehm

**Affiliations:** 1 Department of Gynecology and Obstetrics, Heinrich-Heine University of Düsseldorf, Moorenstr. 5, 40225, Düsseldorf, Germany; 2 Department of Gynecology and Obstetrics, University Hospital of Saarland, Homburg, Germany; 3 Department of Gynecology and Obstetrics, Marienkrankenhaus Hamburg, Hamburg, Germany; 4 Department of Gynecology and Obstetrics, University of Ulm, Ulm, Germany; 5 Department of Gynecologic Oncology, Hannover Medical School, Hannover, Germany; 6 Department of Gynecology and Obstetrics, University of Frankfurt, Frankfurt, Germany; 7 Department of Gynecology and Obstetrics, University of Tuebingen, Tuebingen, Germany; 8 Novartis Oncology, Nuremberg, Germany; 9 Central Academic Hospital, Department of Radiation Oncology, Bielefeld, Germany

**Keywords:** Breast cancer, Bisphosphonates, Zoledronate, Disseminated tumor cells, Survival

## Abstract

**Background:**

The presence of disseminated tumor cells (DTC) in bone marrow (BM) of breast cancer patients is associated with reduced clinical outcome. Bisphosphonate treatment was shown to eradicate DTC from BM in several studies. This controlled randomized open-label multi-center study aimed to investigate the influence of zoledronic acid (ZOL) on DTC and survival of breast cancer patients (Clinical Trial Registration Number: NCT00172068).

**Methods:**

Patients with primary breast cancer and DTC-positive bone marrow were randomized to treatment with ZOL plus adjuvant systemic therapy (n = 40) or adjuvant systemic therapy alone (n = 46) between 03/2002 and 12/2004. DTC were identified by immunocytochemistry using the pancytokeratin antibody A45B/B3 and by cytomorphology. The change in DTC numbers at 12 months and 24 months versus baseline, as well as patient outcomes were evaluated.

**Results:**

86 patients could be included into survival analysis (median follow-up: 88 months, range: 8–108 mths). Patients in the control group were more likely to die during follow-up than those in the ZOL-group (11% vs. 2%, p = 0.106). 15% of patients in the control group presented with relapse whereas only 8% of ZOL group patients developed metastatic or recurrent disease during follow-up (p = 0.205). At 24 months, 16% of patients from the control group were still DTC positive, whereas all patients treated with ZOL became DTC negative (p = 0.032). Patients presenting with persistent DTC 12 months after diagnosis had significantly shorter overall survival (p = 0.011).

**Conclusions:**

Bisphosphonate therapy contributes to eradication of disseminated tumor cells. The positive influence of bisphosphonates on survival in the adjuvant setting may be due to their effects on DTC.

**Trial registration:**

ClinicalTrials.gov Identifier: NCT00172068 [Zoledronic Acid in the Treatment of Breast Cancer With Minimal Residual Disease in the Bone Marrow (MRD-1)].

## Background

The presence of disseminated tumor cells (DTC) in bone marrow (BM) is a common phenomenon observed in 30-40% of primary breast cancer patients. As demonstrated by a large, pooled analysis of BM specimens from more than 4,700 patients, DTC presence at the time of diagnosis is an independent prognostic factor [1]. In addition, it has been shown that tumor cells are able to survive chemotherapy [2] and that their persistence is strongly associated with poor outcome [3]. Therefore, alternative therapeutic options that improve elimination of DTC may reduce the risk of relapse and improve survival in early breast cancer patients.

Recently, two large randomized clinical trials demonstrated zoledronic acid (ZOL) to significantly prolong disease-free survival in breast cancer patients undergoing adjuvant hormonal therapy (ABCSG-12, ZO-FAST). In contrast to these two trials, interim analysis from the AZURE trial did not show a benefit from adding ZOL to adjuvant therapy in the overall patient population [4]. However, subgroup analyses of AZURE data show that ZOL significantly improved survival in patients who were more than 5 years postmenopausal [5,6]. It has been hypothesized that anticancer effects of bisphosphonates (BP) may occur through elimination of DTC from the bone marrow; potential indirect mechanisms of ZOL-mediated activity involve a series of microenvironmental changes, such as angiogenesis inhibition, activation of immune responses, and interactions with mesenchymal stem cells [7-9]. However, limited data are available on the *in vivo* effects of ZOL on DTC.

The aim of the present randomized controlled trial was to evaluate the influence of zoledronic acid on 1) DTC presence after 12 and 24 months of treatment and 2) disease-free and overall survival.

## Methods

### Patients

In total, 96 patients with primary breast cancer were included into this prospective open-label parallel-group study between 2002 – 2004 at four study centers in Germany. All patients presented with minimal residual disease composed of disseminated tumor cells (DTC) in bone marrow at the time of primary surgery and were to receive adjuvant therapy (hormonal, cytotoxic, or both). Exclusion criteria were inflammatory, metastatic, or recurrent breast cancer, creatinine clearance < 30 ml/min, current active dental problems or trauma, or a current or prior diagnosis of osteonecrosis of the jaw (ONJ). Neoadjuvant chemotherapy was not permitted. All patients provided written informed consent prior to initiation of any study-specific procedures. The study was approved by the Ethics Committee at all participating institutions (University of Tuebingen, Hannover Medical School, University of Munich, Klinikum Bielefeld). This clinical study was designed, implemented, and reported in accordance with the international Good Clinical Practice Guidelines with applicable local regulations.

Patients were prospectively stratified on the basis of 6 predefined strata: 2 nodal-status categories (positive, negative) by 3 adjuvant-treatment categories (chemotherapy, hormonal therapy, chemotherapy plus hormonal therapy). Patients were then randomized 1:1 to adjuvant therapy alone (control) or with intravenous zoledronic acid every 4 weeks for 24 months. Per the approved label, zoledronic acid dosing was guided by creatinine clearance.

The primary endpoint was the effect of ZOL on DTC counts after 12 months. Secondary endpoints at 24 months included safety; changes in DTC counts versus baseline; bone-metastasis–free survival, which included death from any cause or bone metastasis; and disease-free survival, which included death from any cause or disease recurrence at any site. Correlative analyses of change in DTC counts with TNM-stage, estrogen- and progesterone-receptor status, and menopause status were planned.

### Detection of disseminated tumor cells in bone marrow

Bone marrow aspirates for DTC assessment were obtained directly before primary breast cancer surgery in all patients. In 71 and 59 cases an additional BM aspiration was conducted 12 months and 24 months after diagnosis, respectively. DTC detection was performed as described in detail previously [10]. 10 to 20 ml bone marrow (BM) was aspirated from the iliac crest into syringes containing heparin anticoagulant under general anesthesia using Jamshidi’s technique. Tumor cell isolation and detection was performed based on the recommendations for standardized tumor cell detection [11]. Samples were separated by density centrifugation using Ficoll (density 1,077 g/ml, Biochrom, Germany). Mononuclear cells were collected from the interphase layer and were spun down onto a glass slide (Hettich cytocentrifuge, Germany) (10^6^ MNC/spot). For detection of cytokeratin-positive (CK) tumor cells, slides were fixed in 4% neutral buffered formalin for 10 minutes and rinsed in PBS. Automatic immunostaining was performed on the DAKO Autostainer using the monoclonal mouse A45-B/B3 antibody (Micromet, Germany) and the DAKO-APAAP detection kit (DakoCytomation, Denmark) according to the manufacturer’s instructions. The A45-B/B3 antibody is directed against common cytokeratin epitopes including the CK heterodimers 8/18 and 8/19. The malignant breast cell line MCF-7 was used as a positive control. For each patient 2 × 10^6^ cells were analyzed on two slides. Analysis was performed on the Automated Cellular Imaging System (ACIS, ChromaVision Medical Systems, San Juan, Capistrano, CA).

### Survival analysis

Follow-up data was obtained from the Cancer Registry of the University of Tuebingen, Germany. For 86 patients a follow-up could be obtained (ten patients were lost to follow-up). Survival analysis was performed by Kaplan–Meier-method. Statistical analysis was performed using SPSS (Version 16) considering p-values less than 0.05 to be statistically significant. Survival intervals were measured from the time of BM aspiration to the time of death or of the first diagnosis of relapse. Relapse was defined as either local recurrence or distant metastasis.

## Results

### Patients’ characteristics

A total of 96 patients were enrolled in the study. Cytokeratin-positive DTC were detected at diagnosis in bone marrow specimens from all patients. The number of DTC ranged from 1 to 35 per 2 × 10^6^ mononuclear cells.

**Figure 1:**
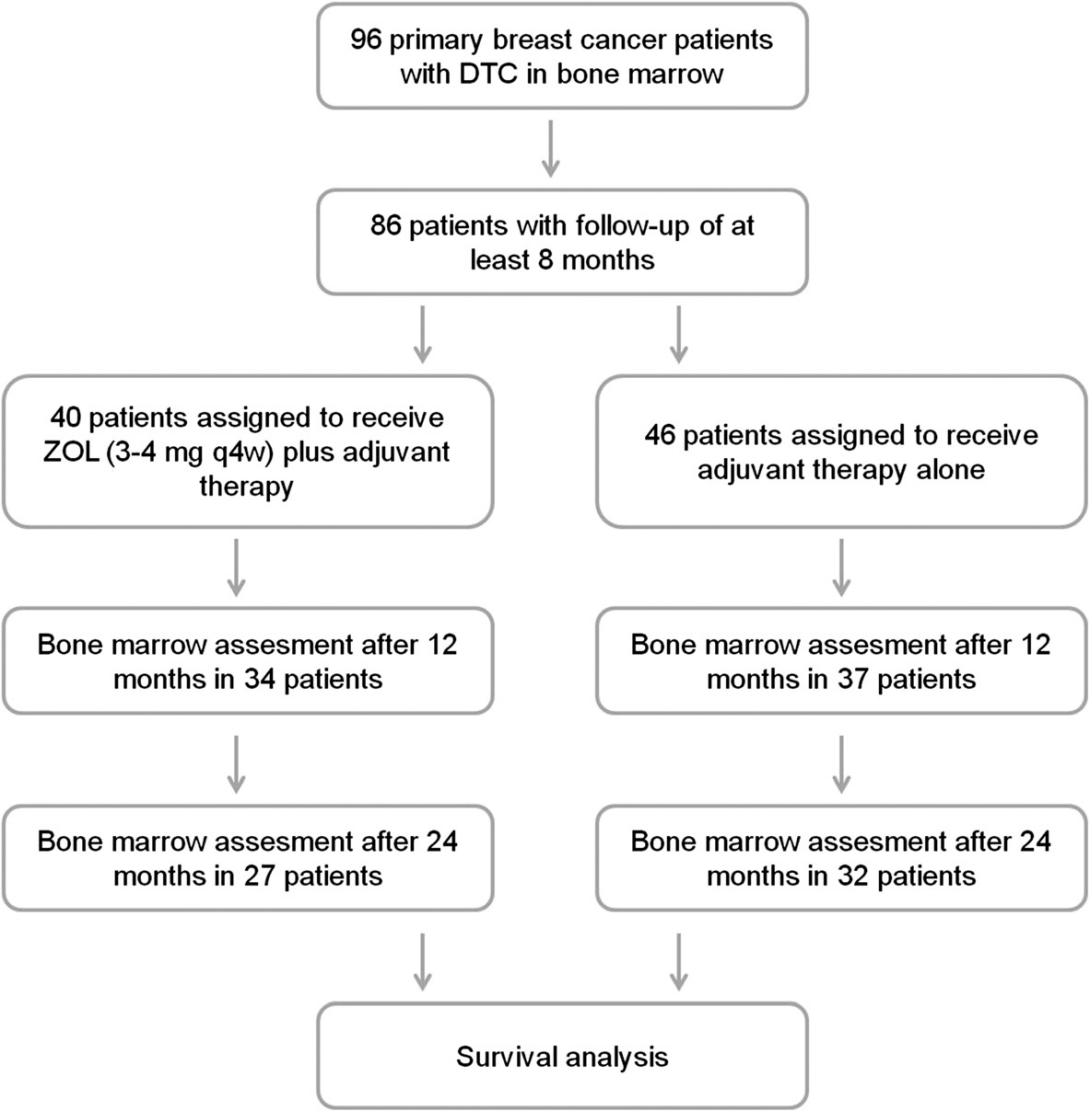
Patient distribution diagram according to the Recommendations for Tumor Marker Prognostic Studies (REMARK).

Patients’ characteristics of all patients were presented previously by Solomayer et al. [12]. In this manuscript we focus on survival analysis. For ten patients no follow-up could be obtained; these patients are censored as lost to follow-up. For 86 patients a follow-up of at least 8 months was available. 64 per cent of these patients had T1 tumors and 88% were node negative. 40 out of 86 patients were assigned to receive adjuvant therapy plus intravenous zoledronic acid every 4 weeks. 46 patients were randomized into control group (adjuvant therapy alone). Clinical data are shown in detail in Table 1. The distribution of patients is summarized in a Recommendations for Tumor Marker Prognostic Studies (REMARK) diagram [13] (Figure 1).

**Table 1:** Clinical data of patients

	**Total**	**ZOL group n (%)**	**Control group n (%)**
Total	86	40	46
Menopausal status			
Premenopausal	55	14 (35%)	17 (37%)
Postmenopausal	31	26 (65%)	29 (63%)
Tumor size			
pT1	51	26 (67%)	25 (61%)
pT2-3	29	13 (33%)	16 (39%)
Nodal status			
Negative	70	35 (90%)	35 (85%)
Positive	10	4 (10%)	6 (15%)
Grading			
I/II	68	33 (87%)	35 (88%)
III	10	5 (13%)	5 (12%)
ER status			
Negative	9	5 (14%)	4 (12%)
Positive	60	30 (86%)	30 (88%)
PR status			
Negative	22	11 (31%)	11 (32%)
Positive	47	24 (69%)	23 (68%)
HER2 status			
Negative	58	30 (86%)	28 (85%)
Positive	10	5 (14%)	5 (15%)
DTC counts at diagnosis Median [range]		2 [1-6]	2 [1-35]

Abbreviations: *BM* bone marrow, *DTC* disseminated tumor cells, *ER* estrogen receptor, *PR* progesterone receptor.

### Survival analysis

86 patients could be included into the survival analysis. The median follow-up of patients was 88 months (range: 8–108 mths). Six deaths were registered during this period. Ten patients (12%) suffered from relapse during follow-up. Eight patients presented with metastatic spread to distant site (three in combination with local recurrence). Two patients were diagnosed with local recurrence only. Data on clinical outcome are summarized in Table 2.

**Table 2:** Survival analysis of 86 patients depending on intravenous administration of zoledronic acid

	**ZOL group n (%)**	**Control group n (%)**
Total	40 (100%)	46 (100%)
Deaths	1 (2%)	5 (11%)
Relapses ^1^	3 (8%)	7 (15%)
Distant metastasis	3 (8%)	5 (11%)
Local recurrence	1 (2%)	4 (9%)

^1^ including local recurrence and distant metastases.

**Figure 2:**
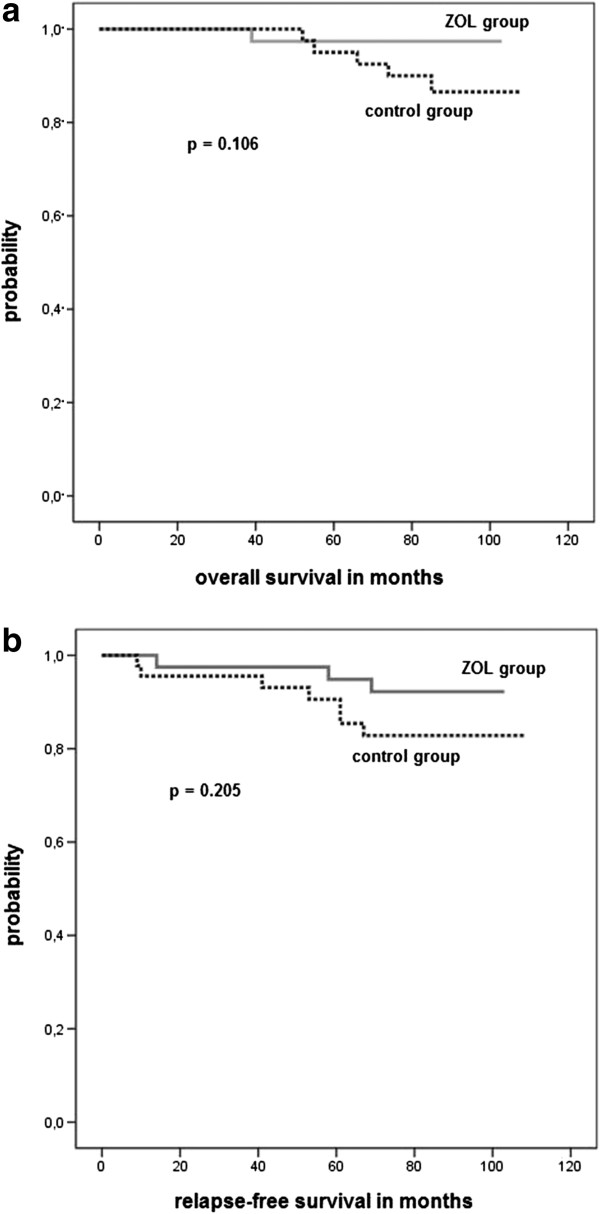
**Survival analysis.** ZOL group – patients assigned to adjuvant therapy plus intravenous zoledronic acid every four weeks. Control group – patients assigned to adjuvant therapy alone **(a)** Overall survival **(b)** Relapse-free survival.

Patients who received adjuvant therapy alone (control group) were more likely to die during follow-up than those who received adjuvant therapy plus zoledronic acid (11% vs. 2%, p = 0.106). In addition 15% of patients in the control group presented with relapse whereas only 8% of ZOL group patients developed metastatic or recurrent disease during follow-up (p = 0.205). These differences did not reach statistic significance due to small sample size. However, a trend toward shorter overall and disease-free survival in the control group was observed. Kaplan-Meier survival curves are presented in Figure 2.

### Persistent disseminated tumor cells at 12 months

**Figure 3:**
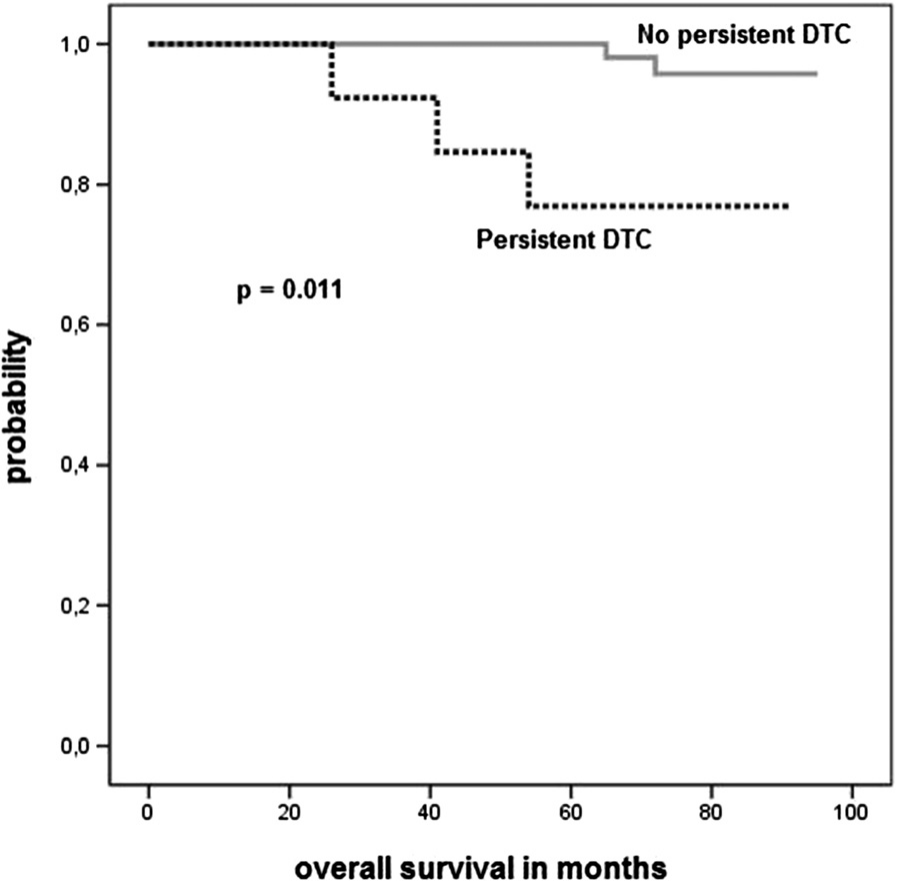
Overall survival analysis with regard to detection of persistent disseminated tumor cells at 12 months after diagnosis.

In 71 cases, a second bone marrow aspiration was performed 12 months after diagnosis. Persistent DTC were detected in 14 out of 71 patients (20%; Table 3). In the ZOL group only four patients were BM positive at 12 months whereas ten patients from the control group failed to clear DTC from their BM (p = 0.106). Patients presenting with persistent DTC 12 months after diagnosis had significantly shorter overall survival (p = 0.011, Figure 3), while disease-free survival remained similar.

**Table 3:** Incidence of persistent disseminated tumor cells 12 and 24 months after diagnosis

	**ZOL group n (%)**	**Control group n (%)**
Second BM aspiration performed after 12 months	34 (100%)	37 (100%)
DTC detected after 12 months	4 (12%)	10 (27%)
Third BM aspiration performed after 24 months	27 (100%)	32 (100%)
DTC detected after 24 months	0 (0%)	5 (16%)

### Persistent DTC at 24 months

**Figure 4:**
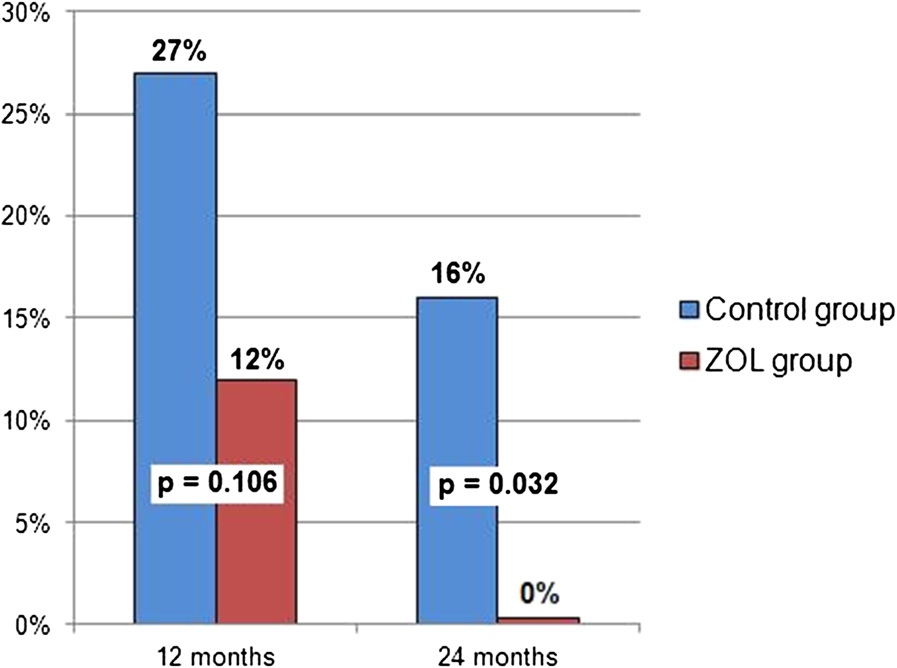
Proportion of patients with DTC-positive bone marrow at 12 and 24 months after diagnosis.

A third bone marrow sample 24 months after diagnosis was obtained in 59 patients. In the control group 5 out of 32 patients (16%) were DTC positive. All patients treated with ZOL became DTC negative 24 months after diagnosis (p = 0.032; Table 3). DTC persistence at 24 months did not correlate with survival. Figure 4 illustrates changes of DTC status in both arms at 12 and 24 months.

## Discussion

This is the first prospective, randomized, controlled study evaluating the influence of zoledronic acid on DTC presence and survival in early-stage breast cancer patients. Our results demonstrate that bisphosphonate therapy contributes to eradication of disseminated tumor cells. All patients treated with ZOL became bone marrow negative after 24 months in comparison to 84% in the control group (p = 0.032). This is in accordance with previous studies; Aft et al. reported that ZOL administered with neoadjuvant chemotherapy resulted in a decreased proportion of DTC-positive patients. This effect did not reach statistic significance (p = 0.054); however, in contrast to our study the reevaluation of bone marrow status was performed after a relatively short time (three months) after begin of ZOL treatment [14]. In the present study no significant correlation between bisphosphonate therapy and clinical outcome could be observed; this is possibly due the relatively small patient number. This is an important limitation of the present trial.

### DTC and microenvironment

Despite progress made in the therapy of breast cancer, the prognosis of patients even with small tumor burden is still limited by metastatic relapse often long after removal of the tumor. Current theories on biology of cancer suggest that disseminated tumor cells detected in bone marrow or blood circulation of patients may serve as a surrogate parameter for minimal residual disease and are therefore precursor of metastasis. However, it is currently not predictable which of these cells will evolve into metastases; presumably, the vast majority undergo apoptosis leaving only a minor percentage able to persist in secondary homing sites [15]. This phenomenon, described as “metastatic inefficiency”, is consistent with the observation that detection of tumor cells in blood or bone marrow does not inevitably cause metastasis; 50% of initially DTC-positive patients do not suffer from a relapse and stay disease-free [1]. Studies suggest that 0.01% of CTC can ultimately produce a single bone metastasis, and at least 10,000 CTC are required for the development of a metastatic colony [16,17]. Nonetheless, approximately one-third of patients fail to clear tumor cells from the blood following removal of primary tumor [18,19]. It is not yet clear, which factors determine the fate of DTC and facilitate their persistence. Recently, oncologic research has focused increasingly not only on the cancer cell itself but on its microenvironment; in this context the concept of a premetastatic niche, permissive to development of (micro-)metastases, has gained much interest [20]. Bone tissue may serve as a suitable microenvironment for the growth of tumor cells derived from the primary tumor in the breast; it represents a dynamic 'habitat’ influenced by a number of parameters, among them angiogenetic mechanisms, osteoclastic bone resorption and hormonal factors [21]. An imbalance between these parameters leads to decreased bone density and osteoporosis; these changes may affect the role of bone marrow as a homing site for DTC. Consequently, bone-targeted agents, such as BP, have not only a stabilizing effect on the bone density itself, but show antitumorigenic activity as well.

### Antitumorigenic effects of bisphosphonates

There is a growing body of evidence of bisphosphonates’ anti-cancer activity *in vivo* and *in vitro*. BP are assumed to alter bone and BM microenvironment affecting its ability to host tumor cells; their effect in the bone is mainly due to inhibition of osteoclast-mediated bone resorption [22]. Several preclinical and animal model-based studies have demonstrated BP to exert direct anti-cancer activity, inhibiting tumor cell adhesion [23], invasion [24], proliferation and angiogenesis [25] and inducing apoptosis [26,27]. Exposure of cortical bone slices to BP partially inhibited breast cancer cell adhesion *in vitro*[23]; interestingly, cancer cells appeared to be more susceptible to BP treatment than normal cells [28]. Boissier et al. demonstrated BP to inhibit breast cancer cell invasion in a dose-dependent manner using a matrigel invasion assay. This effect was partly due to observed reduction in proteolytic activity of matrix metalloproteinases (necessary to digest basement membrane). In that study, ZOL had the highest potency with regard to invasion inhibition, followed by ibandronate, risedronate analogue NE-10244 and clodronate [24]. Wood et al. showed significant antiangiogenic properties of zoledronate *in vitro* and *in vivo*[25]. Further, recent data indicate that nitrogen-containing BP can inhibit the proliferation of human cancer cell lines and induce their apoptosis [26]. Data from animal studies confirm that BP exert potent effects on visceral metastases as well [27,29]. These reports suggest that BP affect the invasive behavior of metastatic cells in secondary sites through both direct and indirect effects and have the ability to interact with tumor cells at each step of the metastatic process.

### Clinical relevance of bisphosphonate treatment in adjuvant setting

The presence of DTC correlates significantly with increased risks of distant metastasis, locoregional recurrence, and death in breast cancer patients [1,30]. Therefore, treatment strategies that target DTC in BM may potentially improve disease-free and overall survival. We hypothesize that bisphosphonates affect the role of bone marrow as a suitable microenvironment for DTCs. Indeed, protective effects of bisphosphonates were reported in clinical trials exploring ZOL as adjuvant therapy in early breast cancer.

Three large prospective studies evaluated the impact of addition of ZOL to systemic treatment on survival (Table 4) [6,8,31]. The randomized open-label Austrian Breast and Colorectal Cancer Study Group (ABCSG)-12 trial (NCT00295646) evaluated the influence of adding ZOL to adjuvant endocrine therapy [31]. In this four-arm trial, 1,803 premenopausal women with hormone receptor positive early stage (stage I-II) breast cancer were randomized to receive goserelin 3.6 mg every 28 days plus either tamoxifen 20 mg daily or anastrozole 1 mg daily, with or without ZOL 4 mg every 6 months for 3 years. At a median follow-up of 62 months, the addition of ZOL reduced risk of disease-free survival events compared with endocrine therapy alone (p = 0.009). The reduction in recurrence was observed locally and distantly both in and outside the bone. The risk of death was also reduced but did not reach statistical significance, although it approached significance in women older than 40 years (p = 0.057).

**Table 4:** Studies evaluating antitumor activity of bisphosphonates in breast cancer

**Study/clinical trial**	**ZO-FAST**	**ABCSG-12**	**GAIN**	**NSABP-B34**	**AZURE**	**Saarto **[32]	**Diel **[33]	**Kristensen **[34]	**Powles **[35]
**N**	1065	1803	3023	3323	3360	299	302	953	1069
**Bisphosphonate**	Zoledronate i.v.	Zoledronate i.v.	Ibandronate p.o.	Clodronate p.o.	Zoledronate i.v.	Clodronate p.o.	Clodronate p.o.	Pamidronate p.o.	Clodronate p.o.
**Duration of therapy**	5 years	3 years	2 years	3 years	5 years	3 years	2 years	4 years	2 years
**Adjuvant therapy**	Endocrine (AI)	Endocrine (Tam vs. AI +GnRH-Anal.)	Dose-dense doseintense CTX	CTX +/- Endocrine	CTX +/- Endocrine	CTX +/- Endocrine	CTX +/- Endocrine	CTX	CTX +/- Endocrine
**Follow up**	60 months	84 months	39 months	91 months	60 months	120 months	103 months	n.d.	66 months
**Premenopausal**	-	Yes (DFS, OS)	No	No	No	No	Yes (OS)	No	Yes (OS, bonemetastasis free survival)
**Postmenopausal**	Yes (DFS)	-	No (Trend > 60 yrs.)	Yes (> 50 yrs. DFS, but not OS)	Yes (DFS, OS)				

In the Zometa-Femara Adjuvant Synergy Trials (Z-FAST/ZO-FAST/E-ZO-FAST), designed to investigate the bone-protective effects of zoledronic acid, an exploratory analysis was conducted to assess the anticancer potential of zoledronic acid. A total of 2,194 postmenopausal women with hormoneresponsive early breast cancer received letrozole 2.5 mg daily [6]. Patients were randomized to receive zoledronic acid 4 mg administered every 6 months for 5 years starting either upon randomization (up-front) or upon a predetermined measure of bone loss (delayed start). After 36 months of follow-up, a 34% reduced incidence of disease-free survival (DFS) events with up-front zoledronic acid treatment compared with delayed treatment was observed in the ZO-FAST trial (*P* = 0.0375). These studies show a DFS benefit or overall survival (OS) benefit in postmenopausal women or women who have chemical ovarian suppression.

In contrast to ABCSG-12 and ZO-FAST trials, the Adjuvant Zoledronic Acid to Reduce Recurrence (AZURE; BIG 01/04) trial (NCT00072020) did not show a benefit from adding bisphosphonates to adjuvant therapy in the overall patient population. The AZURE trial evaluated the antitumor activity of ZOL combined with (neo)adjuvant chemotherapy as well as endocrine therapy in 3,360 pre- and postmenopausal patients with stage II/III breast cancer [4]. Patients were randomly assigned to receive standard adjuvant systemic therapy either with or without ZOL (every 3–4 weeks for 6 doses and then every 3 to 6 months to complete 5 years of treatment). In a second interim analysis, ZOL was not associated with a significant improvement in DFS in the overall population. However, in a subgroup analysis, ZOL improved OS by 29% (p = 0.017) and reduced DFS events in and outside the bone in women who were more than 5 years postmenopausal [8]. Interestingly, in the subset of 195 patients who received neoadjuvant chemotherapy addition of ZOL to neoadjuvant chemotherapy reduced mean residual invasive tumor size by approximately 43% compared with chemotherapy alone. The adjusted mean residuum was 12 mm lower in the ZOL group (15.5 mm) than in the group not receiving ZOL (27.4 mm; p = 0.006), suggesting potential antitumor benefit from combining bisphosphonates with cytotoxic treatment [8].

Further, Aft et al. [14] demonstrated that DTC-free BC patients treated with ZOL (4 mg every 3 weeks) were more likely to remain DTC free at 3 months (*P* = 0.03), and that the subset of patients with estrogen receptor-negative and epidermal growth factor receptor-2–negative disease were more likely to have pathologic complete response with ZOL versus no ZOL. In small studies, ZOL (4 mg/month) increased the proportion of DTC-free patients who remained DTC-free at 6 months versus no ZOL [36], and significantly decreased DTC levels versus baseline at 12 (*P* < 0.0006) and 24 months (*P* = 0.0026) [37] in DTC-positive BC patients. Therefore, it is reasonable to hypothesize that ZOL may delay disease recurrence.

Extensive data on the adjuvant effects of another bisphosphonate, clodronate, were provided by Diel et al. [33]. In that prospective clinical trial 302 patients were randomized to receive either oral clodronate 1600 mg/day for 2 years or standard follow-up. An updated survival analysis with a median follow-up of 103 months showed patients in clodronate group to perform better with regard to OS (p = 0.049).

## Conclusions

Our data confirm the antitumor properties of zoledronate demonstrated in numerous preclinical studies. ZOL treatment effectively eliminated DTC; patients with persistent tumor cells in BM had significantly worse survival. The ability of ZOL to target minimal residual disease may have significant implications for long-term clinical outcome.

## Abbreviations

BM: Bone marrow; BP: Bisphosphonate(s); CK: Cytokeratin; CTC: Circulating tumor cell; DFS: Disease-free survival; DTC: Disseminated tumor cell; ER: Estrogen receptor; HER2: Human epidermal growth factor receptor 2; MRD: Minimal residual disease; ONJ: Osteonecrosis of the jaw; OS: Overall survival; PR: Progesterone receptor.

## Competing interests

WJ has received lecture honoraria for consultation/advisory role from Johnson&Johnson. JH has received lecture honoraria from Novartis, and is an advisory board member for Roche and Novartis. BW is employed by Novartis. MB, EFS, HJL, TF, DW, GG, PH, SB, and BK have declared no conflict of interests.

## Authors’ contributions

MB performed the statistical analysis, participated in the design of the study and drafted the manuscript. EFS, GG, TF, HJL, PH and WJ conceived of the study, and participated in its design and coordination. GG, TF and NK helped to draft the manuscript. NK performed the survival analysis. SB, BK, DW and JH participated in data collection and study coordination. BW coordinated the study and helped to design the study. All authors read and approved the final manuscript.

## Pre-publication history

The pre-publication history for this paper can be accessed here:

http://www.biomedcentral.com/1471-2407/13/480/prepub
